# Comparison of Therapeutic Effects of Topical Calcineurin Inhibitor and Moisturizing Cream on Pruritic External Auditory Canal

**DOI:** 10.3390/jcm10194313

**Published:** 2021-09-22

**Authors:** Sang-Yeon Lee, Soyun Cho, Minju Kim, Dong-Han Lee, Young Ho Kim

**Affiliations:** 1 Department of Otorhinolaryngology—Head and Neck Surgery, Seoul National University Hospital, Seoul National University College of Medicine, Seoul 03080, Korea; maru4843@hanmail.net (S.-Y.L.); auminju@gmail.com (M.K.); 2 Medical Research Center, Sensory Organ Research Institute, Seoul National University, Seoul 03080, Korea; 3 Department of Dermatology, Boramae Medical Center, Seoul National University College of Medicine, Seoul 07061, Korea; sycho@snu.ac.kr; 4 Department of Otorhinolaryngology—Head and Neck Surgery, Konkuk University Medical Center, Seoul 05030, Korea; neocromx@naver.com; 5 Department of Otorhinolaryngology—Head and Neck Surgery, Boramae Medical Center, Seoul National University College of Medicine, Seoul 07061, Korea

**Keywords:** moisturizer, pimecrolimus, pruritic external auditory canal

## Abstract

Although pruritic external auditory canal (PEAC) is a relatively common symptom, particularly in the geriatric population, its pathophysiology and appropriate treatment remain to be elucidated. We compared the therapeutic efficacy of pimecrolimus, a topical calcineurin inhibitor (CI), and a moisturizing cream (MC) in patients with PEAC. Thirty-nine patients (73 ears) were prospectively enrolled and treated topically twice daily with the CI (*n* = 20, 39 ears) or the MC (*n* = 19, 34 ears) for two weeks. The change in itching sensation was evaluated subjectively using a self-questionnaire at immediately, one month, and two months after self-application, and objectively by changes in erythema grading. Although topical treatment with the CI resulted in a more rapid improvement than treatment with the MC in patients with PEAC, the final outcomes did not differ between the groups. Furthermore, similar improvements in erythema scores were noted. The results of this study suggest that the MC, which rejuvenates the normal physiological status of the ear canal skin, may greatly benefit those elderly patients more susceptible to PEAC, without any concerns about adverse events and underlying comorbidities. Expanding upon the understanding of the role of moisturizers in the treatment of pruritic ears merits attention, as this knowledge provides a good example of the clinical guidelines for the management of PEAC.

## 1. Introduction

Pruritic external auditory canal (PEAC), otherwise known as “itchy ear syndrome”, is an isolated itching sensation in the ear canal in the absence of any underlying local or systemic pathology [1]. PEAC is a relatively common otorhinolaryngological symptom found in approximately 4 out of 1000 patients, with a particularly high prevalence in the geriatric population [2].

A relatively acidic pH and hydrophobic milieu in the external auditory canal (EAC), which elicits bacteriostatic properties, is required for the maintenance of a healthy EAC environment [3]. Although the pathophysiology of PEAC has not been clearly elucidated, previous research has suggested that changes in the physiology of the EAC skin contribute to the development of isolated and prolonged itching sensations [4]. Cerumen, a lipid substrate in the EAC, has also been pointed out as a factor that facilitates the homeostasis of the EAC through the antimicrobial effects of substance P and calcitonin gene-related peptide; these molecules of innate immunity may represent key physiological elements in the EAC [5].

Most clinicians would treat PEAC with topical acetic acid (0.25%), with or without hydrocortisone. Although the application of this medication is expeditious in some patients, other patients experience a persistent itching sensation. Previous studies that utilized steroid drops showed positive treatment outcomes in terms of symptom relief [6,7]. However, despite successful results with the use of topical corticosteroids, there remains the risk of skin atrophy, telangiectasia, and a reduction in the keratinocyte count in the EAC skin on a long-term basis [8,9]. Therefore, there is a need to introduce novel, safe, and effective treatments for patients with PEAC.

Recently, a physiological study suggested the possibility of a significant association between EAC hydration and PEAC severity [10]. Given that asteatosis in the EAC, characterized by dry EAC skin and a lack of cerumen, is a common clinical finding in patients with itchy EAC [11], moisturizers that protect the skin barrier could be an alternative treatment for PEAC. Additionally, the use of topical macrolide immunomodulators (i.e., topical calcineurin inhibitors), which are used in the treatment of atopic dermatitis, has been suggested as an appropriate treatment for patients with PEAC [12]. This drug inhibits T cell activation and prevents the release of inflammatory factors from mast cells, and it has a lower side-effect profile compared with corticosteroids [13]. Although this topical agent has been shown to exert significant symptomatic relief in PEAC, there is a lack of evidence surrounding accepted standard treatment strategies for PEAC. Furthermore, the significant gaps in knowledge suggest that it is appropriate to prescribe moisturizers for the treatment of PEAC before considering pharmacological therapy.

Herein, we investigated the clinical efficacy of a topical calcineurin inhibitor and a moisturizing cream as a potential treatment for patients with PEAC. The characteristics of the EAC skin (i.e., subclinical inflammation) and change of symptom with respect to the itching sensation between the two groups are compared, then a possible pathophysiologic mechanism underlying the itching sensation in EAC is discussed.

## 2. Materials and Methods

From January 2018 to December 2020, patients with a chief complaint of itchy ears aged 18 years or older were prospectively enrolled at our medical center. Of them, patients with (1) active bacterial infection including external otitis, external EAC cholesteatoma, and chronic otitis media; (2) evidence of otomycosis; (3) comorbid dermatologic disease (e.g., psoriasis, atopic dermatitis); and (4) a history of canaloplasty or ear canal manipulation, were excluded. The presence or absence of dermatologic disease was evaluated by a board-certified dermatologist (S.C.). Allergen-specific IgE antibody tests were performed to identify patients with specific allergen sensitivities whenever available. Finally, 39 eligible patients (73 ears) with PEAC were enrolled in the study. The patients were randomly classified into two groups and treated topically twice daily with a topical calcineurin inhibitor, a nonsteroidal anti-inflammatory agent (Elidel^®^ cream 1%, Korea Menarini, CI group, *n* = 20, 39 ears), or a nonsteroidal moisturizing cream (Zeroid Soothing Cream^®^, Neopharm, MC group, *n* = 19, 34 ears) for 2 weeks. The present study was approved by the Institutional Review Board (IRB No. 30-2018-31) of our hospital.

**Figure 1:**
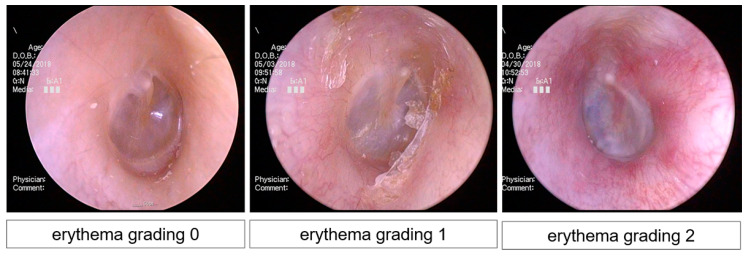
Representative photos used for semi-quantitative scoring of erythema.

At the initial visit, a structured history of the characteristics of PEAC on the affected side and endoscopic findings of the EAC were obtained. All patients completed a comprehensive questionnaire that included a visual analog scale (VAS), medical history of PEAC, and underlying diseases. We assessed the EAC skin in all patients on the affected side during the initial and final visits. Endoscopic findings were captured from locations where the cartilage, bony, and tympanic membranes were completely visible. Subsequently, an experienced dermatologist (S.C.) blindly assigned a grade to all endoscopic findings based on erythema. Erythema (i.e., subclinical inflammation) was classified according to a three-tiered system depending on gross severity (0, negative; 1, weak positive; 2, strong positive) (Figure 1).

Patients were instructed on how to apply the topical agent to the EAC with a cotton-tipped applicator and were asked to do this twice a day for two weeks. To investigate the sustained effect after short-term treatment, the patients were asked to stop the treatment after two weeks, regardless of the treatment response. According to the research plan, the patients were instructed to visit immediately, 1 month, and 2 months after the self-application of each agent for 2 weeks to evaluate the treatment response. The treatment outcomes were classified according to symptomatic relief and maintenance during the follow-up period. In all patients, no additional topical agent or systemic drugs were used for symptomatic relief after the self-application of each agent for 2 weeks during the study period. In this study, post-treatment outcomes were determined using changes in the VAS throughout the follow-up period and subclassified into three groups: “much improved” (50–100% resolution of VAS after 2 months of follow-up relative to baseline), “slightly improved” (0–50% resolution of VAS after 2 months of follow-up relative to baseline), and “stationary” (no change or aggravation). Moreover, the proportion of patients who rebounded (resolution of VAS after 2 weeks of treatment but aggravation during follow-up) was determined in each group.

All data were analyzed using the R statistical package (version 3.3.2; R Foundation for Statistical Computing, Vienna, Austria). All the analyses were performed using GraphPad Prism version 9.0.0 for Windows (GraphPad Software, San Diego, CA, USA). Data are presented as the mean ± standard error of the mean. Demographic differences between the two groups were analyzed using t-tests, Mann–Whitney tests, chi-square tests, and Fischer’s exact tests, as appropriate. An independent t-test or Mann–Whitney U-test was used to compare the VAS and erythema scores between the two groups at specific time points, depending on the distribution of data. Additionally, serial changes in the VAS and erythema scores throughout the follow-up period were analyzed using repeated measures one-way analysis of variance and post-hoc Tukey multiple comparison tests. Statistical significance was set at *p* < 0.05.

## 3. Results

### 3.1. Demographics and Clinical Characteristics

The demographic and clinical characteristics of the participants in each group are shown in Table 1. No significant differences in age at presentation (67.80 ± 2.94 vs. 63.16 ± 2.57, *p* = 0.244 by independent *t*-test), sex (*p* = 0.129 by Chi-square test), positive proportion of MAST (*p* = 0.302 by Fisher’s exact test), or total Ig E titer (*p* = 0.471 by Mann–Whitney test) were observed between the CI and MC groups. None of the patients in either group had experienced systemic diseases that may trigger an itchy sensation, as proposed in our inclusion and exclusion criteria. Detailed profiles of previous treatment modalities for the symptomatic relief of PEAC and underlying diseases are summarized in Table 1.

### 3.2. Subjective Outcomes

**Figure 2:**
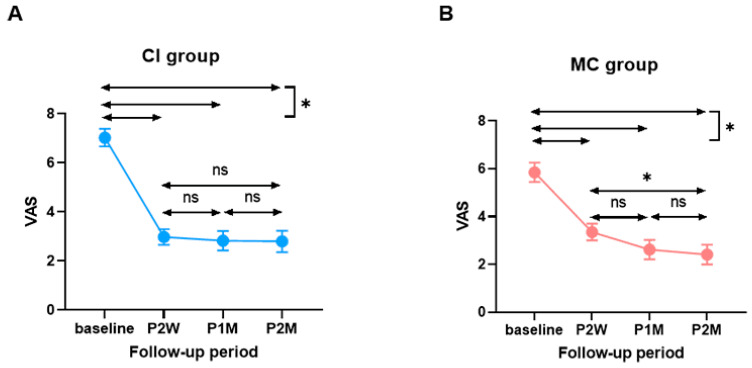
Comparison of subjective improvement of itching sensation over time between the two groups. (**A**) Change of visual analogue scale in the CI group. (**B**) Change of visual analogue scale in the MC group. CI, topical calcineurin inhibitor; MC, moisturizing cream; VAS, visual analog scale; P2W, post-treatment 2 weeks; P1M, post-treatment 1 month; P2M, post-treatment 2 months; *, statistically significant (*p* < 0.05); ns, not significant.

The average pre- and post-treatment subjective symptoms (VAS) in the CI and MC groups are shown in Figure 2. For the CI group, the median pre-treatment VAS score was 7.03 ± 0.36 (range 2–10). The change in the VAS score between the pre- and immediately post-treatment (post-two-week treatment) time points was 4.05 ± 0.38. At the one- and two-month follow-up, the changes in the VAS score were 4.20 ± 0.46 and 4.23 ± 0.47, respectively. In the MC group, the average pre-treatment VAS score was 5.85 ± 0.40 (range 1–10), and the change in the VAS score between the pre-treatment and immediately post-treatment (post two-week treatment) time points was 2.50 ± 0.32. At the one- and two-month follow-up, the changes in the VAS score were 3.23 ± 0.36 and 3.44 ± 0.36, respectively.

**Figure 3:**
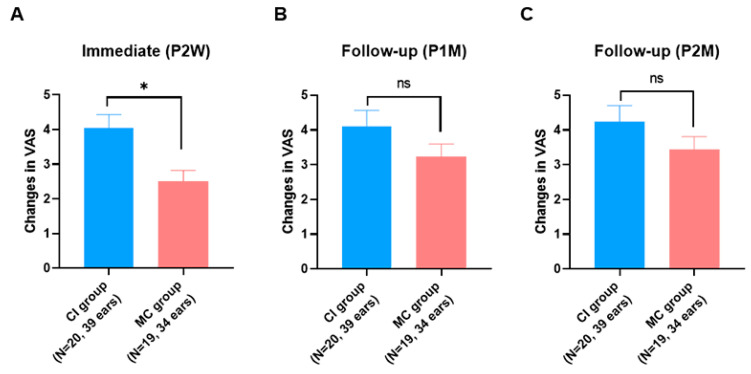
Comparison of subjective improvement of itching sensation at each time point between the two groups. (**A**)Two weeks post treatment. (**B**) One month post-treatment (**C**) Two-months post-treatment. CI, topical calcineurin inhibitor; MC, moisturizing cream; VAS, visual analog scale; P2W, post-treatment two weeks; P1M, post-treatment one month; P2M, post-treatment two months; *, statistically significant (*p* < 0.05); ns, not significant.

Importantly, in the CI group, most of the subjective improvement over the course of the two-month follow-up occurred mainly during the two-week post-treatment period. The VAS scores then remained steady or slowly improved up to two months post-treatment (Figure 2A). Conversely, we identified a slightly different tendency in the MC group. A gradual decrease in the VAS score was noted in the MC group throughout the two-month post-treatment period, while the CI group manifested an abrupt decrease in the VAS score during the early stages (two weeks post-treatment) and then reached a stable status (Figure 2B). In other words, the change in the VAS score during the short-term follow-up (two weeks post-treatment) was significantly higher in the CI group than in the MC group (*p* = 0.0021 by Mann–Whitney test), while the change in the VAS score during the long-term follow-up (two months post-treatment) did not differ between the two groups (Figure 3).

**Figure 4:**
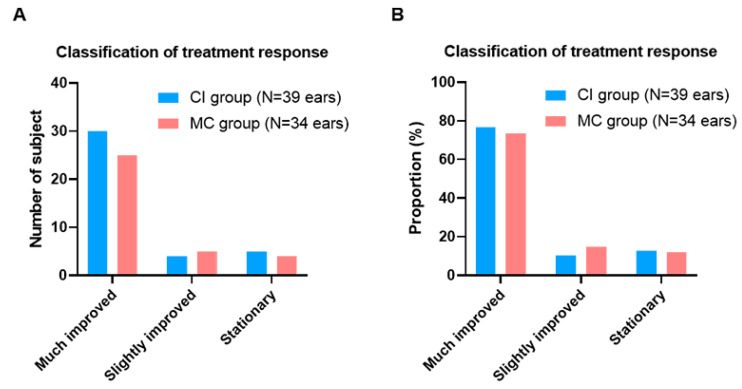
Classification of treatment response with reference to subjective improvement of itching sensation between the two groups. (**A**) Among the 39 ears in the CI group, 30 (76.9%), 4 (10.3%), and 5 (12.8%) met the criteria for “much improved”, “slightly improved”, and “stationary”(**B**) The proportion of patients with “rebound” was 3 (10.0%), 2 (50.0%), and 3 (60.0%) in the “much improved”, “slightly improved”, and “stationary” groups CI, topical calcineurin inhibitor; MC, moisturizing cream.

Among the 39 ears in the CI group, 30 (76.9%), 4 (10.3%), and 5 (12.8%) met the criteria for “much improved”, “slightly improved”, and “stationary”, respectively (Figure 4A). The proportion of patients with “rebound” was 3 (10.0%), 2 (50.0%), and 3 (60.0%) in the “much improved”, “slightly improved”, and “stationary” groups, respectively (Figure 4B). No significant differences were noted in the proportion of types of post-treatment subjective outcomes.

### 3.3. Objective Outcomes

**Figure 5:**
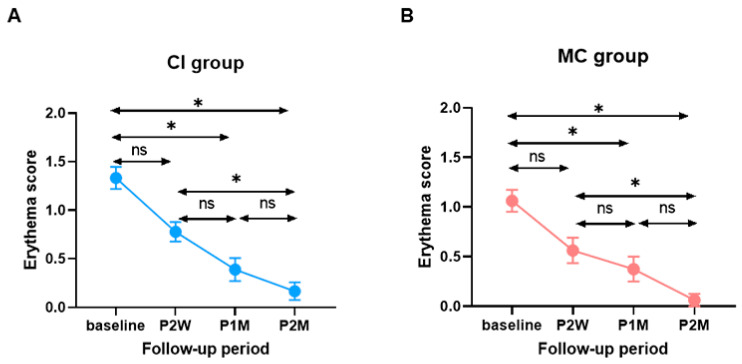
Comparison of erythema score during follow-up periods between the two groups. (**A**) Within CI group. (**B**) Within MC group (*: statistical significance, ns: no statistifcal significance).

The average pre- and post-treatment erythema scores in the CI and MC groups are shown in Figure 5. For the CI group, the average erythema score pre-treatment was 1.33 ± 0.11 (range 1–2). The change in the erythema score between the pre-treatment and two-week post-treatment time points was 4.20 ± 0.46. At the one- and two-month follow-up, the changes in the erythema score were 4.20 ± 0.46 and 4.20 ± 0.46, respectively (Figure 5A). For the MC group, the average pre-treatment erythema score was 1.06 ± 0.11 (range 0–2), and the change in erythema score between the pre- and two-week post-treatment time points was 0.67 ± 0.14. After one- and two-month follow-up, the changes in the erythema score were 0.94 ± 0.13 and 1.17 ± 0.12, respectively (Figure 5B).

Interestingly, a similar improvement in the erythema score was observed between the two groups. A gradual improvement in the erythema score occurred mainly throughout the first month post-treatment and then reached a stable status thereafter. No significant difference in the erythema score was noted between the two groups at any time point.

## 4. Discussion

The current study compared the therapeutic efficacy of PEAC treatments between the CI and MC groups. Both groups showed favorable treatment outcomes following termination of the self-application treatment according to the cross-sectional and longitudinal analyses. Improvement of the itching sensation was demonstrated subjectively based on the VAS, and objectively by changes in the erythema scores via oto-endoscopy. Similar to the significant improvement after CI administration in patients with PEAC, symptomatic relief with MC may be helpful in terms of normalization of the dry, slightly inflamed, itchy skin status in the EAC. The results of this study refine the understanding from previous studies on the management of pruritic ears [2,12,14,15] and provide an additional basis for the clinical guidelines on the use of a moisturizer (i.e., barrier repair cream) for the management of pruritic ears.

Although various treatment options for the relief of pruritic ears have emerged [6,12,16,17], there is no consensus on the accepted standard treatment strategies in the literature. Previous studies that utilized steroid drops or systemic steroids showed positive treatment outcomes in terms of symptom relief [15]. However, despite the successful results, these treatments carry the risk of permanent skin atrophy, telangiectasia, and reduction in the keratinocyte concentration in the EAC skin on a long-term basis [8]. Because the site of pruritus in the ear canal could not be exactly determined, the extensive application of steroids may potentiate the risk of complications, especially in elderly subjects. In line with this, the long-term use of topical steroids has increased the infection rate by Demodex species, which might be a prerequisite for pruritic ears [9]. Given the information from previous studies arguing against the use of topical corticosteroids for pruritic ears, it would be necessary to scrutinize safe and effective pharmacological approaches for PEAC, considering the physiologic perspective of the EAC skin.

In agreement with our results, Djalilian et al. previously suggested the therapeutic effect of CI on itching ears without complications due to chronic use [12]. Specifically, the topical use of CI attenuated the itching sensation in 94% of the subjects, and the production of cerumen was increased by 86%. Although the exact etiologies that cause the itching sensation in the ear canal remain elusive, chronic low-level inflammation associated with aging (termed “inflammaging”) may play a role in PEAC [18]. The deterioration of immune function with age includes a decline in adaptive immunity called immunosenescence, which is accompanied by chronic, low-grade inflammation (i.e., inflammaging) characterized by elevated serum levels of inflammatory cytokines such as IL-6, IL-8, and TNF-α [19]. The accumulation of senescent cells in aging skin reduces its ability to subside inflammation and repair structural damage, which creates a vicious cycle over time. In aged skin, IL-1α secretion was higher in the keratinocytes [20], and Langerhans cells were fewer in number [21]. Langerhans cells are not only important as antigen-presenting cells in the epidermis but are also implicated in the maintenance of the epidermal barrier [22]. Aging also detrimentally affects T cell effector responses in the skin; previous research into the chronic itch of the elderly has indicated a trend toward Th2 cytokine production [12,23]. CI, a second-line treatment for atopic dermatitis, has been reported to inhibit T cell-based immunity by suppressing the transcription of early cytokines and preventing the release of inflammatory substances from mast cells [24,25]. Furthermore, skin penetration and absorption of CI were 70–110 times lower than those of corticosteroids [26]. Therefore, it can be tolerated by thin-natural skin, especially in the EAC [27], for prolonged periods with a low side-effect profile. Collectively, as observed herein, CI might reduce the inflammatory response and elicit an antimicrobial environment, possibly leading to the improvement of the itching sensation and erythema in the EAC. Further research regarding immune-mediated inflammatory conditions after CI treatment would be needed.

Herein, we longitudinally followed up our cohort and monitored the itching sensation and erythema scores immediately after the termination of the self-application treatment and at up to three time points throughout two months, which has not been achieved in many studies. This thorough evaluation at several time points, including in the early stages immediately after termination of the two-week treatment, enabled us to speculate on the working mechanism related to various pharmacological approaches. Intriguingly, our data showed that subjects treated with CI experienced an abrupt decrease in itching sensation mainly in the early stages, reached a plateau at two weeks, and became relatively stable thereafter for up to two months. In contrast, a gradual improvement in itching sensation was noted with the MC, resulting in a temporary, significantly less subjective improvement compared with the improvement achieved using the CI at two weeks post-treatment. However, this gradual decrease in itching sensation with the MC became especially prominent from two weeks to two months post-treatment, leading to the loss of significant differences at one and two months after treatment. These results suggest that the pharmacological properties and working mechanism of therapeutic agents for pruritic ears could affect the symptomatic relief in different ways.

Nevertheless, both groups showed overall favorable treatment outcomes, with similar improvement in itching sensation and erythema score, from two weeks to two months post-treatment. The proportion of subclassification profiles based on the subjective improvements did not show any statistically significant differences between the two groups. Furthermore, a similar tendency toward improvement in the erythema score was noted, regardless of the pharmacological approach. Therefore, we need to speculate how the MC elicits therapeutic outcomes comparable to those of the CI.

Theoretically, the normal aging process may lead to further deterioration of the protective layer of the thin EAC skin and exacerbate the secretory functions of the glands [4,24,28]. In support of this evidence, Kurban et al. showed that along the aging process, the size of the glands in the EAC tends to increase, while the secretory function decreases [28]. These age-dependent structural and functional alterations in the EAC skin may impair cutaneous immune and inflammatory responses, resulting in an itching sensation. Moreover, the dryness of the EAC skin tends to cause itching and irritation [27]. Tuzuner et al. demonstrated that the treatment effect may be associated with the moisture level of the EAC skin, suggesting that alterations in the normal EAC physiology may be imperative in the pathogenesis of itching in the EAC [10]. Previous studies have shown that the use of moisturizers can prevent trans-epidermal water loss from dry, slightly inflamed, itchy skin, and offer symptomatic relief. For example, the use of ceramide-containing moisturizers has been shown to improve pruritus, xerosis, and quality of life in elderly subjects with atopic dermatitis [24]. Considering that elderly subjects have an increased susceptibility to pharmacological treatment side effects, the use of moisturizers that normalize the skin barrier function would be optimal before considering pharmacological therapy [24]. MC, a physiological lipid-containing skin moisturizer, contains ingredients that protect and strengthen the skin barrier. Furthermore, one of these ingredients stimulates the production of antimicrobial peptides, thus reinforcing the functions of the skin barrier and the self-defense system. Collectively, if the skin barrier condition in the EAC is suboptimal, an itching sensation may occur, especially in elderly patients. In this case, MC, a physiological lipid mixture, was able to restore the normal physiological status of the EAC skin and clinically improve the itching sensation.

Expanding upon the understanding of the role of moisturizers in the treatment of pruritic ears merits special attention, as this knowledge provides a good example of the clinical guidelines for the management of PEAC. The results of this study may greatly benefit elderly patients with PEAC and could be useful in mitigating adverse events, underlying comorbidities, and physiological changes that occur in the elderly. Despite the encouraging data, this study has some limitations that warrant future follow-up studies. First, the small sample size may have weakened its clinical implications, albeit in a prospective manner. In addition, data on longitudinal changes over the two months after treatment are needed to support the sustainability of both modalities. Alternatively, further data on the treatment response after long-term treatment (more than two weeks) are necessary, as they may contribute to an optimal strategy for treating PEAC. Second, in terms of the itching sensation, only the VAS scores could be adopted over time, thus providing limited information concerning the subjective symptom improvements. Finally, objective methods for evaluating symptom changes are needed. Although the changes in erythema and desquamation in response to the treatment can be documented, the objective evaluation using oto-endoscopy is inherently limited by its subjective nature. Currently, the degree of inflammation or the moisture level of the EAC skin is putatively correlated with the itching severity and treatment outcomes [2,10]. An objective test that evaluates the physiological characteristics of the EAC skin, including sebum level, pH, and hydration, or histologic analysis from the skin biopsy would provide a better understanding of the action mechanisms of the CI and MC, coupled with subjective improvements. Hence, future prospective and large-scale longitudinal follow-up studies with additional subjective questionnaires and more accurate objective methods should be performed to prove the clinical effectiveness of the CI and MC, and to better understand the pathophysiological mechanism of pruritic ears. Lastly, further evidence of an immune-mediated inflammatory component in PEAC may refine our clinical data and open a new path for treating PEAC, leading to better outcomes and compliance.

## 5. Conclusions

We, for the first time, compared the therapeutic efficacy of PEAC between the CI and MC. Similar treatment outcomes, with respect to the itching sensation and erythema score, were noted. The results of this study suggest possible pathophysiologic mechanisms underlying the itching sensation in EAC. Collectively, MC that rejuvenates the normal physiological status of the ear canal skin may greatly benefit elderly patients more susceptible to PEAC, without raising any concerns about adverse events and underlying comorbidities. Expanding upon the understanding of the role of moisturizers in the treatment of pruritic ears merits attention, as this knowledge provides a good example of the clinical guidelines for the management of PEAC.

## Figures and Tables

**Table 1:** Demographics and clinical characteristics in patients with pruritic external auditory canal.

Topical Calcineurine Inhibitor Group (*n* = 20, 39 Ears)																
Subject	Age	Sex	Laterality	Culture	Total Ig E	MAST	Underlying Disease	Previous Itchy Ear Treatment	Intial VAS (R)	Intial VAS (L)	P2W VAS (R)	P2W VAS (L)	P1M VAS (R)	P1M VAS (L)	P2M VAS (R)	P2M VAS (L)
1	69	M	B	NG	40.8	Negative	HTN	Steroid otic drop	10	10	5	5	2	2	2	2
2	46	M	B	NG	961	DF (class 2), Cladosporium (class 1)	Angina	Antihistamine (oral)	7	4	1	1	0	0	0	0
3	65	M	B	NG	20.5	DF (class1)	-	Steroid otic drop	4	6	4	6	6	7	7	7
4	64	F	B	NG	17	Negative	HTN	-	10	10	3	3	1	1	2	1
5	81	M	B	NG	<2	Negative	Angina	Vinegar irrigation	5	5	2	2	2	2	2	2
6	90	M	L	NG	7.63	Negative	HTN, BPH	-	0	7	0	6	0	6	0	6
7	80	F	B	NG	21.7	Negative	Dementia	-	8	10	2	1	2	1	2	1
8	85	F	B	NG	12.3	Negative	-	Antibiotics Otic drop	8	5	5	3	3	3	2	2
9	74	M	B	NG	236	Negative	-	-	6	6	2	1	1	1	0	0
10	49	F	B	NG	42.8	DP (class 1)	-	-	5	5	3	3	0	0	0	0
11	68	M	B	NG	84.9	Negative	HTN, Arrythmia	Topical steroid oint	10	10	1	1	3	3	2	2
12	52	F	B	NG	130	Positive control > 100	-	-	6	2	2	0	6	1	6	1
13	61	F	B	NG	393	DP/DF (class 2/3)	HTN	-	5	8	3	6	2	2	2	2
14	57	F	B	NG	36	Negative	HTN	Topical steroid oint	10	10	3	3	4	4	7	7
15	64	F	B	NG	<2	Positive control > 100	HTN, DM (on insulin)	-	8	5	5	0	5	0	5	0
16	78	M	B	NG	1160	DF (class 2)	HTN	-	5	5	2	2	2	2	2	2
17	45	F	B	NG	22.3	Negative	HTN	Antihistamine (oral)	8	7	2	1	1	2	1	1
18	79	F	B	NG	31.8	Japanese hop (class 1)	HTN	-	6	6	2	2	3	3	3	3
19	78	M	B	NG	10	Negative	BPH	Topical steroid oint	10	10	8	8	10	10	10	10
20	71	M	B	NG	160.7	Negative	HTN	Vinegar irrigation	6	6	4	3	4	3	3	2
**Topical moisturizing cream group (*n* = 19, 34 ears)**																
**Subject**	**Age**	**Sex**	**Laterality**	**Culture**	**Total Ig E**	**MAST**	**Underlying** **disease**	**Previous** **itchy ear ** **treatment**	**intial VAS** **(Rt.)**	**intial VAS** **(Lt.)**	**P2W VAS** **(Rt.)**	**P2W VAS (Lt.)**	**P1M VAS** **(Rt.)**	**P1M VAS (Lt.)**	**P2M VAS** **(Rt.)**	**P2M VAS** **(Lt.)**
1	54	M	L	NG	39.1	Negative	HTN, DM (on insulin)	Antihistamine (oral)	NA	9	0	3	0	4	0	4
2	63	F	B	NG	23.9	Negative	Ovarian cancer	-	5	7	2	5	1	2	1	2
3	82	M	B	NG	<2	Negative	HTN	Steroid otic drop	9	1	9	1	9	1	9	1
4	58	F	L	NG	4.84	Negative	-	-	0	6	0	5	0	0	0	0
5	55	F	B	NG	10.8	DF (class 1)	-	-	5	5	3	3	2	2	1	1
6	69	F	B	NG	53.2	Negative	-	Topical steroid oint	3	3	1	1	0	0	0	0
7	31	M	B	NG	102	cat (class 4) dog (class 2)	-	Antibiotics otic drop Antihistamine (oral)	7	7	7	7	7	7	7	7
8	64	F	B	NG	115	DP/DF (class 1/1)	-	Steroid otic drop	10	10	4	4	6	6	6	6
9	63	F	B	NG	94.3	Negative	-	-	5	5	3	3	1	1	1	1
10	69	M	B	NG	<2	Negative	HTN	Vinegar irrigation	2	4	1	3	1	1	1	1
11	55	F	B	NG	18.3	Negative	HTN	-	5	5	2	2	4	4	4	4
12	64	F	R	NG	9.97	Negative	HTN	Topical steroid oint	8	0	2	0	0	0	0	0
13	63	F	B	NG	85.1	Negative	HTN	-	4	8	3	5	3	4	2	2
14	59	F	R	NG	59.7	Negative	-	Topical steroid oint	8	0	5	0	6	0	6	0
15	70	F	B	NG	3.32	Negative	-		2	7	0	4	0	1	0	0
16	75	F	B	NG	21.8	Negative	-	Steroid otic drop	6	6	4	4	3	3	2	2
17	57	F	B	NG	29	Negative	-	-	3	6	1	3	1	3	1	3
18	80	M	B	NG	83.9	Negative	HTN, BPH, Angina	-	4	8	3	7	1	1	1	2
19	69	F	B	NG	19.5	DP/DF (class 1/1)	HTN	Vinegar irrigation Antihistamine (oral)	7	9	1	3	1	3	1	3

Abbreviation: M, male; F, female; R, right; L, left; B, both; NG, no growth; Ig E, Immunoglobulin E; MAST, multiple allergen simultaneous test; VAS, visual analog scale; HTN, hypertension; DM, diabetes mellitus; BPH, benign prostate hyperplasia; P2W, postoperative 2 weeks; P1M, postoperative 1 month; P2M, postoperative 2 months.
